# Emotional upheaval, the essence of anticipatory grief in mothers of children with life threatening illnesses: a qualitative study

**DOI:** 10.1186/s40359-022-00904-7

**Published:** 2022-08-11

**Authors:** Kazem Najafi, Azam Shirinabadi Farahani, Maryam Rassouli, Hamid Alavi Majd, Maryam Karami

**Affiliations:** 1 Student Research Committee, Departmant of Medical-Surgical Nursing, School of Nursing and Midwifery, Shahid Beheshti University of Medical Sciences, Tehran, Iran; 2 Department of Pediatric Nursing, School of Nursing and Midwifery, Shahid Beheshti University of Medical Sciences, Tehran, Iran; 3 Department of Biostatistics, School of Allied Medical Sciences, Shahid Beheshti University of Medical Sciences, Tehran, Iran

**Keywords:** Anticipatory mourning, Emotional upheaval, Mother, Life-threatening illness, Child

## Abstract

**Introduction:**

Life-threatening illnesses in childhood are considered a crisis for the whole family, especially for the mother, which leads to experiencing different degrees of grief and emotional-behavioral problems.

**Objective:**

The present study is conducted with the aim of explaining the concept of anticipatory grief from the perspective of the mothers of the children with life-threatening illnesses.

**Methods:**

This qualitative study is part of a sequential exploratory research for model development in the field of anticipatory grief, which was conducted using conventional content analysis method. The data were collected through in-depth semi-structured interviews with 19 mothers of the children with life-threatening illnesses living in Shiraz and Tehran, who were selected through purposive sampling with maximum variation. When data saturation were achieved, the data were codified by MAXQDA-10 software and analyzed using Graneheim and Lundman’s approach.

**Findings:**

Data analysis resulted in the identification of 8 subcategories including shock, irritability, fear of losing, feeling guilty, uncertainty, hopelessness, loneliness and isolation, and mourning without a coffin, all forming the major theme emotional upheaval.

**Discussion and conclusion:**

As the result of being in a situation of imminent and possible loss of her child, the mother experiences different responses of sadness and sorrow and suffers the consequences the core of which is consisted of emotional upheaval. Therefore, by explaining the behaviors related to anticipatory grief, efficient and effective interventions can be designed to improve coping among this group of mothers.

## Introduction

Millions of children worldwide live with life-threatening conditions caused by chronic diseases. The number of children with life-limiting conditions (LLC) and life-threatening illnesses (LTI) is increasing globally [1], which is considered a challenge for healthcare professionals and parents [2]. In a study conducted in 2011, using the concept of death trajectory and the codes of the 19th Revision the International Classification of Diseases (ICD-19), Randall et al. [3] have classified children’s life-threatening and life limiting conditions into 4 categories: the gradual and progressive decline in health and moving towards death (e.g. neuromuscular dystrophy), recurring periods of intensive care to maintain the quality of life (e.g. cystic fibrosis), curative treatments which may fail (e.g. cancer), and severe but non-progressive disability along with extreme health vulnerability (e.g. spastic quadriplegia with tracheostomy). This sub-group of children is the most fragile from the medical point of view and they have several features in common such as extensive use of health care services, polypharmacy and other health care interventions to maintain the quality of life, the risk of frequent and long-term hospitalization. Therefore, there is no reasonable hope for their treatment and possible death awaits them [4].

Despite major advances concerning life-threatening illnesses, the prognosis, life expectancy, and the quality of life among these children is still unclear. The development of a life-threatening illness in one child can lead to a crisis for the whole family and impact its life, where parents consider themselves responsible for the child’s illness and experience anxiety, the feeling of guilt, frustration and helplessness, which is why family caregivers have been called hidden patients [5, 6]. There are different descriptions regarding the loss of a child, a spouse, a sibling, a parent, a close friend, or an acquaintance. However, it has been said that the loss of a child is the most painful and long-lasting compared to other losses, because it is against the laws of nature that parents are outlive their children [7]. Besides, recent evidence has shown that the mothers with a dying child suffer from the symptoms of traumatic stress and experience more severe problems along with emotional disorders in comparison with what may happen due to the death of a spouse. The reason is that, unlike in the past, when families might have several children, death in childhood may be experienced as the parents’ future dreams ruining as well as a profound change in their current roles and functioning, due to greater attachments to only children [8]. Other studies have also shown that when a child develops a threatening illness, the mother is more involved in providing care for the child than the father, and takes up more responsibility regarding decision-making and treatment. Therefore, she experiences more changes in her life, and because of spending more time with her child, she suffers from higher mental tensions such as denial, anxiety, depression, uncertainty, and the fear of her child’s death, which limits her ability to address her own and other family members’ needs, causing her to feel she has lost the primary control over life [9, 10]. Each of these emotional reactions is considered as the main indicator of anticipatory grief [11].

Anticipatory grief is a phenomenon consisting of the phases mourning, coping, interaction, planning, and psychological reorganization in response to the imminent loss of a loved one [12]. Kumar and Nyatsuro [13] define anticipatory grief as an active process of sadness that occurs before the actual loss. During this period of waiting for death, the mother begins to mourn and experiences different responses of sadness and sorrow. The concept of anticipatory grief was first introduced by Lindemann [14] in 1940 as a safeguard against the effects of a sudden loss that facilitates adjustment through mourning. Since then, the concept of anticipatory grief has received much attention from researchers. Anticipatory grief is a potential opportunity for preventive intervention with the aim of minimizing the preventable effects of sadness and sorrow. The experience of anticipatory grief can be helpful, for one has time to prepare and learn coping skills for inevitable changes. This phenomenon, especially in palliative care, is an inseparable part of the process of sadness and sorrow [15]. The results of some studies also showed that even though the parents know that their child’s disease is incurable, they do not give up on their child. They use all kinds of treatments, which are mostly ineffective, to prolong their child’s life. Therefore, to help reduce their discomfort following these failures, they use every opportunity to strengthen their love and attachment to their child, since they think that it can partially compensate for the grief of losing the child in the future [16].

However, unlike the results of the previous studies, in the study by Ounalli et al. [17] it was found that when a child develops a life-threatening illness, parents use long-term strategies to cope with imminent death. They try to adapt to the shock and hopelessness and, while preparing for the possible death of their child, help their child experience less pain and endure it. Under these circumstances, the development of anticipatory grief leads to functional fatigue and psychological maladjustment and causes family caregivers to experience high levels of mental distress. In general, the specific problems stemming from the possible loss of a child feel somehow like “losing a limb”, the clear sense of permanent loss of a part of yourself that you may adapt to, but will not grow back [8].

Therefore, by considering various aspects and definitions of the concept of anticipatory grief in different studies, and achieving a better understanding of this phenomenon with focus on the cultural context, we aim to specifically examine the manifestation of the loss experienced during the child’s life, to reduce the problems caused by maladjustment through identifying the concepts affecting anticipatory grief and providing preventive and up-to-date coping strategies and interventions, and to take effective measures for the emotional management of these mothers.

## Methods

The current qualitative study was conducted using the conventional content analysis approach to explain the concept of anticipatory grief from the perspective of the mothers of the children with life-threatening illnesses considering the Iranian cultural context.

### Participants

The participants were the mothers visiting Shahid Beheshti University of Medical Sciences in Tehran (Shohada-ye Tajrish, Mofid, Imam Hossein and Loghman hospitals) and Shiraz University of Medical Sciences (Namazi and Amir Hospitals) whose child had a life-threatening disease like cancer, cardiovascular, pulmonary diseases, severe cerebral palsy, and muscular dystrophy. All the participants were Muslims. The inclusion criteria consisted of being at least 18 years old, living in the same place with her husband and her 1 to 16-year-old sick child, having an appropriate mental health, and being in charge of care provision for the sick child. The mental health status of mothers was evaluated by taking history and self-report. The participants were selected through purposive sampling with maximum variation in terms of demographic variables such as age, socioeconomic status, educational level and the place of residence. The sample size was determined to be 19, according to data saturation. Therefore, sampling continued until data saturation were achieved and no new code emerged [18].

### Data collection

The data were mainly collected through semi-structured in-depth interviews using open-ended questions and making field notes. In-depth interviews were used for discovering and explaining the meaning of the studied phenomena [19]. The sampling was performed from August to September 2021. Initially, the mothers’ characteristics were collected according to their child’s medical records. They were then visited or telephoned. After explaining the objectives of the study and obtaining consents, the appropriate time for the interview was agreed upon. All the mothers’ children had been hospitalized or recently discharged. As requested by the participants, the interviews were conducted separately in a private and quiet room, such as the lounge of the inpatient ward, in accordance with the ethical principles. The interviews were conducted by the first author in Persian language and subsequently translated to English. After that, the manuscript sent to a native English editor to ensure that the standards of the academic language would be guaranteed in the translation process. In some cases where in-person interviews at home were possible neither for the researchers nor for the participants due to COVID-19 pandemic, data were collected through in-depth semi-structured telephone interviews or video-chats via WhatsApp. An electronic recorder was used to record all the interviews. The Family Caregiver Anticipatory Grief Clinical Interview Scale (FcAG-CI) was used to select the interview items [20]. The interviews began with opening questions related to anticipatory grief such as “How do you feel about your child’s illness?”, “How has this disease changed your life?”, “What has been the most difficult aspect of care provision?”, “How do you deal with it?”, “Have you ever felt like you were losing your child?”, “How did you feel when this happened to you?”, and “What do you think might happen in the future?” Follow-up and exploratory questions were then asked to elucidate the topic at hand and to cover research objectives. The interviews lasted between 30 and 90 min. No participants refused to participate or withdrew after giving their consent. The researcher also made observational and field notes and used memo writing.

### Data analysis

Data collection and analysis were done simultaneously. The data were analyzed using Graneheim and Lundman’s conventional content analysis approach. Immediately after each interview, it was written down word by word, and the text was reviewed and read several times with the aim of obtaining a general understanding of the content. During the data analysis process, at first, the whole text of each interview was considered as an analysis unit whose meaning units were groups of words or sentences which gave an identical meaning or were relevant to the same concept in some ways. Next the primary codes that were the result of condensed meaning units were extracted and were named and classified based on their similarities. Through ongoing comparison of primary classes based on similarities, differences, and proportions, abstraction was performed. Finally, the main themes representing the mother’s understanding were extracted. It is worth noting that the data were codified using MAXQDA-10 software [21].

The following statement is as an example of how data were coded:

“Only I could understand and enjoy combing her hair and caressing her and her body covered in bruises and injection scars which smelled of infection. No one understands what I have gone through,” said participant No. 16, which is mentioned here as an example. This statement was considered as a meaning unit and its primary condensed unit was the lack of understanding of the unique and complicated situation of the illness by others. This code was categorized in the subcategory factors affecting mother’s loneliness.

### Trustworthiness

In this study, in order to evaluate the accuracy and the quality of the findings, Guba and Lincoln’s four criteria of trustworthiness were used including credibility, dependability, confirmability, and transferability [22]. To ensure the credibility of the data after analysis, each interview was returned to the participants and the accuracy of the content was confirmed and the necessary changes were made. To observe the unbiased interpretation and to identify the confirmability, the coding process was performed by all authors and compared with each other. Lastly, the disagreements were resolved by consensus. Moreover, In order to enhance the confirmability, the research steps, its methodology, and the decisions made at various stages were elaborated on, so that, if necessary, other researchers could track the research. To ensure dependability, the codes extracted from each interview were peer checked by the colleagues and experts of this field, and to improve transferability, the mothers were chosen who were as different as possible in terms of demographic characteristics.

## Findings

According to the findings of the present study, the mothers were facing a unique mental phenomenon and experience known emotional upheaval due to a sudden and traumatic change caused by their child’s illness prior to the actual loss, which was accompanied by a wave of unpleasant emotions and leading them into an unusual state from which they could not escape. It caused them to regard their experience as transformation. During this incident, their senses were involved and their energy was depleted, while mentally trying to overcome, and they showed signs of psychological maladjustment. Therefore, this concept was considered as the main point of anticipatory grief due to its extent, scope, and recurrence. The average age of mothers was thirty-six years old. Table 1 shows the demographic characteristics of the research participants. By analyzing the data, the main theme emotional upheaval was formed with the subcategories shock, Irritability, fear of loss, feeling of guilt, uncertainty, hopelessness, isolation and loneliness, and mourning without a coffin (Table 2).

**Table 1:** The demographic characteristics of the mothers participating in the research

Participant no.	Age (year)	Occupation	Socioeconomic status	Level of education	Child’s age (year)	Child’s illness	Time elapsed since diagnosis (months)
1	28	Housewife	Poor	High school diploma	2	Muscular dystrophy	6
2	36	Housewife	Med	Bachelor’s degree	7	Cancer	30
3	32	Employee	Med	Master’s degree	10.2	Cancer	12
4	29	Employee	Med	Bachelor’s degree	3	Congenital heart defect	16
5	31	University professor	Good	PhD	1.5	Cerebral palsy	8
6	43	University student	Poor	Bachelor’s degree	3	Spinal muscular atrophy	24
7	38	Teacher	Med	Bachelor’s degree	4.5	Chronic kidney failure	13
8	26	Licensed practical nurse	Med	Associate’s degree	8	Brain tumor	36
9	25	Housewife	Poor	High school diploma	4.5	Cancer	10
10	35	Housewife	Poor	Master’s degree	6	Cancer	24
11	36	Self-employed	Good	Bachelor’s degree	6.5	Cystic fibrosis	60
12	42	Self-employed	Poor	High school diploma	9	Metabolic syndrome	9
13	49	Self-employed	Poor	Secondary school	2.5	Spina bifida, Hydrocephalus	30
14	37	Housewife	Med	High school diploma	3.7	Pulmonary agenesis	18
15	42	Employee	Med	Bachelor’s degree	2.8	Congenital heart defect	24
16	35	University student	Med	Master’s degree	5.6	Cancer	16
17	54	Housewife	Poor	High school diploma	16	Cancer	132
18	27	Housewife	Poor	Bachelor’s degree	12	Cancer	12
19	40	Employee	Med	Bachelor’s degree	7.8	Cancer	48

**Table 2:** Categories and subcategories of the main theme “Emotional upheaval, the Essence of Anticipatory Grief”

Main theme	Categories	Subcategories	Condensed units
Emotional upheaval	Immediate reactions	Shock	The taboo of life-threatening illness, the trauma that changes the whole life in an instant, wishing that the current situation were just a dream, the inability to understand the meaning and spiritual distress
		Irritability	Not tolerating uncertainty, being angry with the illness, an excuse to release one’s inner pressure, poor quality services provided by the health system, medical staff’s being inconsiderate, the heavy burden of care, the changes in family responsibilities and roles
	Delayed reactions (emotional scar)	Fear of loss	Fear of losing the sick child, fear of losing other children
		Feeling of guilt	Blaming oneself for not providing proper care, cultural beliefs regarding mother’s atonement for her sins
		Uncertainty	Inability to explain the meaning of illness-related events, the child’s unclear future, inability to predict illness outcomes
		Hopelessness	Feeling of worthlessness, being unmotivated, false delight
		Loneliness and isolation	Others’ lack of understanding of their unique condition, withdrawal from social activities to prevent the deterioration of complications, depression, emotional frigidity, crying and wailing, sense of not belonging, an opportunity to pray, the lack of social supportive resources
		Mourning without coffin	Bidding frequent unanswered farewells to the child, early embracing of mourning ceremonies and rituals, the hope of reunion in the afterlife, surrendering to fate and destiny

### Immediate reactions

The life-threatening and unexpected nature of the disease, at the time of diagnosis or a change in the child’s condition, led to emotional and uncontrollable reactions such as shock and irritability in most mothers.

#### Shock

From the very beginning, all the mothers experienced a feeling of disbelief. The main factor that played an important role in the shock was the fear of the name of illness, reflecting the taboo of life-threatening illness, and the mothers’ poor understanding of the term diagnosed (such as cancer, cerebral palsy) for their child.

“After my baby was born, we were shocked by the diagnosis and I started crying. I even saw my father crying. I had never seen Papa’s tears. Oh, my God…” (Participant No. 10)

Even mothers with a family history of the disease described it as a trauma that had changed their entire lives.

“Everything is strange and unbelievable for me and my family. Everything changed in the blink of an eye. I wish someone would wake me up and say it were all a dream” (Participant No. 12)

The participants could not have imagined that their child would suffer from such adverse condition at such a young age, and it was very difficult for them to accept. It is possible that difficulties with meaning and in fact the spiritual distress contribute to mothers’ surprise and perplexity.

“Why should this have happened to a boy who is only four years old, while there are so many adults and elder people?” (Participant No. 9)

#### Irritability

The feelings of rage were tangible in various dimensions such as anger towards the current situation and illness, and being angry with medical experts and especially physicians, the inability to tolerate uncertainty, the heavy burden of care, the changes in family roles and responsibilities, and the reduced quality of life. The participants did not know how to deal with these reactions when faced with anger and felt bad about their behavior towards the family members and friends.

“I must be able to have more control over myself, so I do not have to constantly yell at my husband and children and lose my temper easily,” (Participant No. 12)

Because of losing peace and patience, some mothers became irritable and angry with those around them, releasing their inner pressure.

“My elder daughter complains and says mom just picks on us without any reason, to release her inner pressure and be free of pain.” (Participant No. 14)

Many of these mothers reported poor quality health care services, stating that health care providers are reckless and insensitive and knew them as one of the causes of own irritability and anger.

“On the first day and without preparing us, the doctor told us that our son is diagnosed with cancer. It’s extremely awful, and although I said I seek God’s help… I am really angry with them. They could have broken the bad news in another way.” (Participant No. 18)

### Delayed reactions (emotional scar)

During and after the treatment, the subjects experienced disturbing scenarios when confronted with the reminders and the flashbacks of the moment of diagnosis and treatment, resulting in emotional reactions and challenges such as the fear of loss, the feeling of guilt, uncertainty, hopelessness, loneliness, and mourning without a coffin, which can be described as an emotional scar or an emotional consequence.

#### Fear of loss

The participants felt fear and anxiety as they imagined a world without their beloved one. The mothers unanimously expressed grief and concerns about the child’s future and the stress of losing their child.

“How many more times do we have to do the injections and infusion therapy? I’m afraid of losing my child due to injections and infusions.” (Participant No. 2)

“Sometimes I suddenly wake up sweaty in the middle of the night after having a nightmare of my beloved one’s funeral.” (Participant No. 19)

Due to the nature of the illness, the parents felt compelled to live with the bitter reality that they would sooner or later lose their child. Many mothers believed that they should not live longer than their children.

“At first, I did not believe at all that my child had such a disease, but as he got worse, thoughts of losing him constantly came to my mind, and I could not sleep at night because of such worries and concerns. I have asked God many times to take my life instead of his.” (Participant No. 8)

The fear of loss was so intense in some participants that made them feel that their other children might suffer from a serious, undiagnosable, and worrying illness that could threaten their lives and future, too, while in fact they had no serious problem or illness.

“I always think that my two other children have cancer, too, even though they have been examined and the results are negative. But I still do not believe it. When they get a simple headache, I become too scared.” (Participant No. 17)

And they finally acknowledged that early death was inevitable, unfair, and painful, and that the thoughts of losing their child came to their minds every day. They were afraid of the moment of definitive loss.

“We were always thinking of the disease, like a family whose child is a murderer, every morning expecting for his death sentence to be executed.” (Participant No. 7)

#### The feeling of guilt

While coping with the disease, the participants repeatedly experienced the feelings of guilt and remorse for their previous actions, such as timely visit and examination. Some mothers even associated the cause of the disease with their time of pregnancy.

“It is all my fault that my child is in such a condition now. I did not take good care of him, especially during my pregnancy.” (Participant No. 9)

According to some cultural beliefs that consider any difficulties and illnesses as atonement for their sins in this world, the mothers had thoughts in this regard and reviewed their previous sins.

“I stay up until midnight and wonder endlessly, which of my sins is my child paying for?” (Participant No, 15)

#### Uncertainty

An important mental stressor that affected the lives of most of the participants in many aspects was the inability to discover the meaning of disease-related events or the inability to predict the consequences of the disease. Due to the complexities of the treatment and the symptoms, these mothers experience a high level of uncertainty that is caused by their inability to perform daily tasks, inadequate treatment, and concerns about the child’s future.

The following excerpts and quotes reflect the uncertainty which is an evident sign of emotional upheaval.

“This period is exactly like moving on the edge of the precipice for us. You cannot predict what will happen. Sometimes, one physician gives us hope and, sometimes, another ruins our hopes.” (Participant No. 4)

“Everything is uncertain. We never really feel at peace. I always think about the disease, that symptoms like the shortness of breath may come back, and the stuff….” (Participant No. 14)

Sometimes everything is going well, from the test results to the general state, but again there are moments when everything goes wrong.” (Participant No. 2)

“We do not know how long we are going to stay in this prison? One year? Ten years?” (Participant No. 1)

“We have visited several physicians so far. There are many inconsistencies in their opinions. Each of them gives a different answer. We are frustrated.” (Participant No. 11)

“I want to know what will happen to him in the next three years. Can he adapt to the community? Can he go to school?” (Participant No. 9)

#### Hopelessness

The mothers expected that their children would grow up moving towards a bright future. When they encountered a disease in which the experience of growing up meant early death, it made them not only sad, but also helpless and hopeless.

“We used to like to have several children. Since this condition emerged, we would choose not to have children again. We were totally disappointed.” (Participant No. 11)

All the participants expressed, in different ways, their experiences of grief and sorrow during care, the intensity of which varied during the care period, but never went away completely.

“These days I live with tears in my eyes, I’m stuck in a life of grief, totally sunk in despair.” (Participant No. 2)

Although hopelessness was not reported in the diagnosis phase, progressive hopelessness was experienced over time, during the course of treatment.

“It is normal that you experience a positive feeling when someone talks to you about good things. But all I have heard is that everything is bad and my child will not survive. Now, those words are gradually being proven to me.” (Participant No. 4)

Many mothers expressed their helplessness and frustration in providing adequate care, and the root of some of this sorrow and hopelessness was their inability to solve the problem and the fact that despite making much effort they could not take an effective step towards treating their child.

“My child is fading away before my eyes, and I have no power to do anything for him.” (Participant No. 16)

Some mothers felt hopeless because they saw that the treatments were in vain and lost their strength without getting any results.

“At least, I think that giving these drugs to my child has no effect, and I am thinking about treatment with traditional medicine, that may be better.” (Participant No. 17)

Some participants stated that sometimes feelings of hopelessness are so strong that they have suicidal ideation.

“Maybe the cure for my pain would be suicide, if only I were not afraid of the punishment in the afterlife.” (Participant No. 8)

#### Loneliness and isolation

During many interviews, the feeling of loneliness was evident from several different aspects.

The complicated situation of these children is so unique that it cannot be easily understood by those who have not experienced it before, and this made the main caregiver, i.e. the mother, feel lonely and isolated.

“Only I could understand and enjoy combing her hair and caressing her and her bruised body covered in bruises and injection scars which smelt of infection. No one understands what I have gone through.” (Participant, No. 16)

Loneliness and isolation as being one possible consequence of all the time needed to attend to the child which is exacerbated by social factors. They spent nearly their whole time with their children and gradually withdrew from social activities to prevent the deterioration of complications and, as the disease progressed, became increasingly isolated.

“The disease and the medications had weakened the immune system so much that we were afraid of even a simple infection. Sometimes, we did not spend time with our friends and acquaintances for several months, even with my parents, especially during autumn and winter. It was very difficult for us.” (Participant No. 5)

One of the factors that led to the sense of loneliness was insecure attachment, and part of the concern was due to the fact that they were not happy and saw the world as a gloomy place. Even their partner (spouse) could not stay with them during the time. Although people around them might try to lighten up their mood, these mothers themselves were reluctant to communicate in order not to cause troubles for their relatives and acquaintances, and sometimes preferred to talk to God; therefore, silence, isolation, and loneliness was necessary for praying to God.

“All I try to do is to keep my feelings to myself. This way I do less harm to my husband and my family, and they undergo less pressure. Therefore, I held my tears in.” (Participant No. 5)

Many interviews also showed a feeling of impatience and bad mood.

“I always visited my relatives and thought about them. But honestly, I’m not in the mood right now. I’ve been isolating myself for a while.” (Participant 1)

Low quantity and poor quality of social support resources, and barriers against access to services and facilities in the community, and the lack of awareness on available services had also exacerbated this situation, and mothers considered themselves as unsupported.

“I feel completely miserable when I, as a rural woman, have to sit in this hospital without any financial support and refuge and look at the tall, soulless buildings of the city through the window of my room,” (Participant No. 17 in tears)

They believed that medical staff were not committed to homecare, and complained that the health care system could not understand their living conditions. Therefore, these mothers had to deal with the death of their children alone.

“It is very unfortunate that no one cares for or understands us when we are discharged,” (Participant No. 13)

### Mourning without a coffin

The results of this study showed that mothers who provide care for a child with a life-threatening condition often realize at the time of diagnosis that their child will not live long and that he/she will probably pass away before they do, beginning the grief process long before the actual death of the child and embracing a set of behaviors and thoughts that indicate the imminent loss of their child. This subcategory has deep roots in typical Iranian burial customs.

“I keep thinking about the last time he says goodbye to me. *Goodbye mom! Tonight may be the last time I see you*.” (Participant No. 18)

“I know that one of these days he/she will fly, but I will be seeing him/her again in no time. The difference is, then, we would never leave each other ever again.” (Participant No. 10)

The participants accepted the fact that the presence of their child in this world is accompanied by a lot of suffering, and despite their inner desire, had no choice but to bear it and surrender to the imminent death of their child, while seeking shelter in God and leaving it all to Him.

“I won’t probably ever see his wedding night, and I do not know what part of God’s plan this is, losing him so soon.” (Participant No. 9)

## Discussion

The participants consisted of the Iranian mothers whose children had a variety of life-threatening diseases, from cancer to congenital heart defect and neurological conditions. The findings from theme analysis showed that the experience of anticipatory grief for a mother with traumatic distress is caused by exposure to the child’s life-threatening condition, and this separation distress challenges the participants for a long time in their efforts to regulate emotional feelings.

The experience of anticipatory grief for the mothers in this study was a wave of unpleasant emotions and an emotional upheaval that began with the diagnosis of the disease and continued until the actual loss. During this time, the lifestyle was changing to leave more time to provide care for and spend time with the child. Not only was the experience inseparable from emotions, it was completely intertwined with it. During this transition period, the emotions were related to the fact that the dying loved one would no longer be present in this world. When this transition from illness to death was perceived by the mother, this awareness was expressed by psychological emotions such as shock, the lack of self-control, the fear of loss, the feeling of guilt, uncertainty, hopelessness, loneliness, and isolation, and mourning without a coffin. The participants’ expression of feelings in this study was consistent with the results of many studies, and it shows that emotional upheaval is the nature and basis of anticipatory grief. The study by Coelho et al. [12] showed that anticipating death causes problems at several levels, where family members feel that their reality is constantly affected by new and disturbing events, and that the whole world is shaken. The relationship with the patient and the family structure changes and soon all life will inevitably undergo changes.

The devastating shock felt after learning about the child’s condition at the first moments of diagnosis was a term frequently referred to by the mothers. One of the main reasons for this shock and disbelief was breaking bad news without preparation and showing empathy by the medical staff, especially the doctors. These mothers expected the medical staff to choose the words carefully and to use appropriate words such as “serious illness” instead of “cancer” or “intense and long-term treatment” instead of “chemotherapy”, because for Iranians diseases such as AIDS and cancer are considered as no return and no cure points [23]. In addition, the guidelines for breaking bad news recommend the forewarnings that reduce shock and ease the emotional burden, and consider it necessary for health care workers to predict a wide range of grief responses and even encourage the expression of feelings of sorrow and grief [24].

In this study, irritability was observed due to not tolerating uncertainty, the heavy burden of care, the changes in family roles and responsibilities, and the reduced quality of life appeared as rational or irrational anger. These reactions were triggered as a transient emotion and a way to ease the present emotional failures. In the study by Mooney-Doyle et al. [25] on the challenges experienced by the parents of the children with LTI, the situation was described as a *battlefield* that triggered verbal and non-verbal anger and rage. Most of the mothers participating in this study considered the connection with the source of hope and God’s essence as a determining factor for achieving peace. The findings of several other studies also showed that due to the fact that the majority of the society are Muslims, in the Iranian culture, religious confrontation is the most common method used by the parents of the children with life-threatening diseases to control the adversities and tragedies of life [26, 27]. They try to find meaning and place an external center of control over the situation in order to achieve peace [26, 28]. Furthermore, helping to recall memories with the aim of redefinition and cognitive reconstruction by replacing negative thoughts with positive ones and strengthening the child-mother relationship will cause emotional discharge, and yields control over irrational behaviors [29].

One of the extracted themes was the mother’s concerns regarding the thoughts of death and the fear of losing the child, a subject that had constantly occupied their minds and depleted their energy. In a systematic review study conducted to synthesize the recent research with the aim of developing more knowledge on the family experience of anticipatory grief, the results showed that for most family caregivers in Western societies, due to anticipating the death and sudden loss of a loved one and suddenly recalling painful scenes, anticipatory is a very frightening, stressful, and confusing experience which causes distraction. While the patient is physically present and in need of care, the mother does not have the usual functioning and is unable to deny her grief [30]. In Popejoy’s study [31] in regard with parents’ experiences of care decisions for the children with life-limiting illnesses, the parents’ initial response after a definitive diagnosis was panic and fear due to the fact that they associated the illness with imminent death. Pishkouhi et al. [32] considered the feeling of losing a child as the parents’ biggest and worst problem. Dutta et al. [33] also stated that all the participants in their study spoke of their absolute lack of hope in their child’s survival, and because of their negative perception, they associated their child’s illness with death, all consistent with the results of the current study.

In this study, the concept of the feeling of guilt was formed due to the fact that many mothers considered themselves responsible for the current situation of the child, so they blamed themselves and felt guilty. Yang et al. [16] also reported that when children’s illness begins to deteriorate and family members realize that their child’s condition is beyond their control, they feel guilty and think they may have something to do with their child’s current condition. In the study by Gómez-Zúñiga et al. [34], one of the most prevalent emotions among the home care givers of the children with rare diseases was the feeling of guilt. Caregivers, especially mothers, have always felt worried about neglecting their child and have tried to make up for it by providing more care. The result of this behavior was maternal burnout and fatigue as a caregiver. In the present study, another possible reason for the mothers’ feeling of guilt was associated with their cultural and religious beliefs. They believed that any difficulty and disease is a response to human sins in this world and they searched for the roots of their sins. The results of the study by Nikfarid et al. [27] also pointed to this cultural belief. In their study, they concluded that since Muslims believe in “supernatural causes of diseases”, they often seek to find religious reasons for everything which they suffer from. They search for a refuge for themselves while dealing with negative and stressful events through performing religious and spiritual rituals such as avoiding sins, fasting, saying prayers, making vows, and feeling the need to visit the tombs of Shia Muslim imams in Mashhad (a holy city for Muslims in Iran) and Karbala (a holy city for Muslims in Iraq). It is important to mention that the ultimate goal of Islam is to encourage individuals to move towards transcendence and spiritual advancement. However, it is obvious that the lack of awareness on the part of some mothers, especially the less educated ones, who only rely on general beliefs regarding religious matters, can make the process of adapting to a child’s chronic illness more complicated and lead to negative results. For example, the results of Abu-Raya et al.’s [35] study showed that the use of some religious coping strategies such as “redefining God’s power to influence a stressful situation” and “begging God for a miracle” are associated with more negative consequences on mental health and spiritual distress.

The daily challenges of experiencing special care, worries about the child’s uncertain future, and disagreements between medical team members and parents were among the main factors of this research that indicated uncertainty. The results of the study by Olson [36] showed that the caregivers of the patients with chronic life-threatening illnesses such as cancer have an uneven path ahead with multiple possibilities. In addition to the expected anticipatory grief, these companions experience indefinite loss and grief due various reasons such as unclear and uncertain therapeutic future and being forced to make decisions based on limited information, which in line with the results of the present study, shows that this uncertainty results in nothing but emotional distress.

According to the findings of the present study, the participants experienced hopelessness accompanied by sorrow and grief both for themselves and for the dying child, which was a deep feeling. Their voice went down while describing the grief they felt. Most of them cried while expressing this common emotional experience, and believed that their condition is not fair. A similar pattern of reactions was observed in the study of Fox et al. [37] and Cheung et al. [38].

Most of the mothers in this study reported a feeling of loneliness after the diagnosis. This finding contradicted the results of the study by Al-Gamal and Long [11] on the parents of children with cancer, where the results showed that after diagnosis, their relationship with people around them had improved, and the family foundation had strengthened. The feeling of loneliness may persist even in the physical presence of others [39]. According to the participants, the reason for this isolation and loneliness was the fact that either those around them could not understand their feelings and concerns, or the mother herself preferred to talk to God during these critical conditions, so she require silence and loneliness. Another reason was that they did not have social supportive resources. In an exploratory study by Lauren Breen et al. [40] which solely focused on caregivers’ preparation for the death of their loved ones, it was concluded that although the caregivers were cognitively or behaviorally prepared, they were not emotionally prepared and spent their time in loneliness and isolation, because of being overwhelmed by the heavy burden of care without any support during the course of an unpredictable illness.Therefore, given that predicting the possibility of the child’s death under such circumstances has impacts on emotional energy and concentration, avoidant, and anxious attachment will result in a more intense and overwhelming experience of anticipatory grief, while secure attachment will lead to higher levels of adjustment and coping with grief in mothers [41].

Mourning without a coffin was another extracted subcategory that caused emotional upheaval and distress, and was engaged in the process of anticipatory grief. The participants of this study accepted the fact that their child would not have a long life, and that his/her presence in this world was associated with a lot of pain. Thus, despite their inner desire and based on the principle of beneficence, they had no other choice than to accept and surrender to the imminent death of their child. As they sought shelter in God and left the outcome to Him, they prepared themselves in advance to mourn and embrace a series of unpleasant behaviors and thoughts. This only resulted in mental distress (i.e., depression and emotional fatigue) and higher levels of anticipatory grief. However, other studies on grief confirm that discussing religious and traditional rites and ceremonies helps parents maintain an ongoing relationship with the dying child [42]. This inconsistent result may be due to the fact that in the traditional Iranian culture, talking about death is a taboo [43] and none of the knowledgeable relatives and experts discussed the topic with the mother, so the mother herself had dealt with it alone and mostly in an extreme way. Therefore, by focusing on family and social relationships, grief counseling can play an important role in providing end-of-life support. In this regard, the study by Dutta et al. [33] recommends that medical staff use new mental, social, and spiritual interventions such as the Family Dignity Intervention (FDI), which aim to promote palliative care. Through Meaningful and conceptual interviews, the FDI approach can facilitate mutual interaction for parents, develop an understanding of rational strategies, promote coping with each other in response to the child’s death, and create a common ground for parents and their families to better support each other.

In addition, according to Kübler-Ross’s theory, the type of one’s emotional reaction regarding the death of a loved one and its acceptance depends entirely on one’s actions, behavior, thoughts, personality structure, and ability to cope with past issues and problems in life [44]. It can be confidently said that the medical staff’s knowledge, especially nurses, of anticipatory grief as a phenomenon of cultural nature, as well as their familiarity with care needs and personality and psychological characteristics of people, leads to effective interventions for improving adjustment among this group of mothers [45]. The advantage of this study is that it was performed in the central and referral hospitals of Tehran, as the capital of Iran, and Shiraz, the medical hub of south of Iran where peoples with different cultures come to these centers from all over the country. However, this study was carried out among few groups of Iranians, while there are several subcultures in Iran that differ in terms of culture, lifestyle, ethnicity and language. In order to eliminate the impact of this problem, in future studies, participants can be selected from different provinces with diverse cultural backgrounds. In addition, although the results of the study reveal the experiences of mother caregivers, they may not be generalizable due to the qualitative nature of the research and nonrandom sampling approach.

## Conclusion

The findings of this study show that being in the transition phase of anticipatory grief leads to specific behaviors and processes. The anticipatory-grief-related processes identified in this study included caregiving challenges accompanied with intense emotional reactions. Reactions that led to inappropriate behavior under normal circumstances, the lack of control over emotions, inability to establish or maintain satisfactory interpersonal relationships, and unpleasant moods or depression. In addition, considering that caregiver’s anticipatory grief is related to many factors such as mother–child attachment bond and care burden, and some of the needs and aspects that are unique to the anticipatory grief in this group of mothers might be overlooked; So there is a need to develop anticipatory grief guidance which provides appropriate information and support the expression of feelings. In other words, to meet the needs of these families and manage emotional upheaval, health institutions can get help from different types of coping strategies such as education and information, spiritual care model of sound heart, remembrance activities, follow-up contact, establishing keepsakes and therapeutic writing.

Finally, the study revealed that social support is a crucial factor affecting the exhausting experiences of anticipatory grief. Hence, social support from the closed ones, health-care professionals, as well as from the community, would be valuable in coping with the anticipatory grief.

## Data Availability

Due to privacy concerns, the transcripts of the interviews are not available to the public. On reasonable request, the corresponding author can provide transcript information.

## Abbreviations

LLC: Life-limiting conditions
LTI: Life-threatening illnesses
AG: Anticipatory grief
FcAG-CI: Family Caregiver Anticipatory Grief Clinical Interview
